# Editorial: The role of exosomes in neuroinflammation and neurodegeneration

**DOI:** 10.3389/fncel.2022.1109885

**Published:** 2022-12-23

**Authors:** Yi Wang, Xiaohuan Xia

**Affiliations:** ^1^Translational Research Center, Shanghai Yangzhi Rehabilitation Hospital Affiliated to Tongji University School of Medicine, Shanghai, China; ^2^Center for Translational Neurodegeneration and Regenerative Therapy, Tongji Hospital Affiliated to Tongji University School of Medicine, Shanghai, China; ^3^Center for Translational Neurodegeneration and Regenerative Therapy, Shanghai Tenth People's Hospital Affiliated to Tongji University School of Medicine, Shanghai, China; ^4^Shanghai Frontiers Science Center of Nanocatalytic Medicine, Tongji University, Shanghai, China

**Keywords:** neuroinflammation, neurodegeneration, extracellular vesicle, astrocyte, microglia, exosome

Neuroinflammation, driven by immune cells including microglia and astrocyte, have emerged as a key contributor of the pathogenesis of various neurological diseases. Activated immune cells release neurotoxic and inflammatory molecules and extracellular vesicles including exosomes to promote the formation of neuroinflammatory microenvironment, which leads to neural damage and neurodegeneration. Exosomes are small bilipid layer-enclosed vesicles that can be secreted by all types of brain cells. Being a key intercellular communicator, exosomes have been demonstrated as an important component of neuroinflammatory microenvironment, that regulates various pathological processes through delivery of bioactive cargos within the central nervous system (CNS) (Xia et al., 2019, 2022; Kalluri and LeBleu, 2020). Recent studies have unveiled the significant contribution of exosomes to neuroinflammation and neurodegeneration in acute brain damage such as stroke and chronic neurodegenerative diseases like Alzheimer's disease (AD) (Heneka et al., 2014; Dugger and Dickson, 2017; Xia et al., 2019, 2022). In this topic, Li P. et al. reported that neuronal cell line N2a's autophagy moderately increased in response to heat stress and accelerated by microglia cell line BV2 cells *via* the transfer of exosomes to neurons. They identified that microglial exosomal miR-155 is the key regulator of heat stress-induced neuronal autophagy as evidenced by loss-of-function and gain-of-function interventions. Furthermore, they found that Rheb is a functional target of miR-155 and that microglial exosomal miR-155 accelerated heat stress-induced neuronal autophagy mainly by regulating the Rheb-mTOR signaling pathway, suggesting that exosomal miR-155 could be a promising target for interventions of neuronal autophagy after heat stroke. Wu et al. examined immune cells and exosomal transcriptome regulatory networks in patients with methamphetamine use disorders (MUDs) undergoing withdrawal. They reported a significant decrease in CD3^+^T and CD4^+^T cell numbers in MUDs patients undergoing acute withdrawal, and that the diminishment was restored to baseline in patients undergoing protracted withdrawal. Interestingly, the differentially expressed exosomal mRNAs/long non-coding RNAs in MUDs patients undergoing withdrawal were significantly enriched in neurodegeneration-related diseases. Likewise, Wang et al. observed differential expression patterns of circulating exosomal miRNAs from the CSF and blood of Alzheimer's disease (AD) patients, identified the predicted targets of these differentially expressed circulating exosomal miRNAs, and analyzed their biological functions and interactions. Their results showed the temporal regulation of complex signaling networks on AD pathogenesis and shed light on the development of novel therapeutic strategies with multi-target drug combination for AD treatment.

On the other hand, exosomes with therapeutic effects have been suggested as potential therapeutic agents or drug delivery platforms for the treatment of neurological diseases. In this topic, Li N. et al. utilized cellular and mouse AD models to investigate the therapeutic effects and underlying mechanism of M2 microglia-derived exosomes (M2-EXOs) on AD progression. They found that M2-EXO treatment increased cell viability, restored the destruction of mitochondrial membrane potential, and reduced the accumulation of reactive oxygen species inside the mitochondria and cells in a dose-dependent manner in an AD cell model. Moreover, they reported that M2-EXOs internalized by HT-22 cells and MAP2-positive neurons in APP/PS1 mice exerted protective effects as evidenced by reduced Aβ plaque deposition and decreased Aβ oligomer expression, *via* enhancing PINK1/Parkin pathway-mediated autophagy. These results indicate a protective role of M2-EXOs in AD onset and progression, suggesting M2-EXOs as a novel therapeutic approach for AD. Yang et al. reviewed the mechanism of action of exosomes obtained from different cell sources in the treatment of spinal cord injury (SCI) and the regulatory role and therapeutic potential of exosomal non-coding RNAs. They also discuss future opportunities and challenges and propose that exosomes and exosomal non-coding RNAs might be promising tools for the treatment of SCI.

Exosomes derived from specific cells types exert particular functional roles in the brain. In this topic, researchers discussed roles of glial cells-secreted exosomes. Glial cells include astrocytes, microglia, and oligodendrocytes. Exosomes from these cells modulate intercellular communications in the brain and exert protective or neurotoxic effects on neurons. Liu et al. and Oyarce et al. reviewed roles of exosomes derived from glial cells. Liu et al. highlighted recent research progresses on astrocytes-derived exosomes and summarized the roles of GABAceptive and GABAergic astrocytes that serve as an inhibitory node in CNS intercellular communications. Oyarce et al. summarized the state of the art on how exosomes secreted by glial cells affect neurons and other glial cells in CNS during neuroinflammation and neurodegeneration, and on how specific stress stimuli and pathological conditions change the bulk levels and properties of glial cells-secreted exosomes.

To summarize, recent studies have demonstrated a tight association of exosomes with neuroinflammation and neurodegeneration through the direct delivery of pathogenic molecules, the modulation of inflammatory responses of immune cells, the regulation of neuronal cell function and viability, and so forth. The altered contents of exosomes under pathological conditions provide novel perspectives to achieve early diagnosis for neurodegenerative diseases, the key to effective prevention of irreversible neuronal loss. Moreover, drugs targeting pathogenic exosomes as well as exosomes with therapeutic effects or drug delivery capacity have demonstrated promising therapeutic potential in alleviating behavioral and pathological phenotypes of neurological diseases in cell and animal models. With extensive investigations, more pathological/beneficial roles of exosomes in neuroinflammation and neurodegeneration will be unveiled to improve our understanding of exosomes and shed light on the development of exosome-based therapeutic strategies for more precise diagnosis and more effective treatment of NDs.

## Author contributions

Both authors listed have made a substantial, direct, and intellectual contribution to the work and approved it for publication.
